# Attitude Disparity and Worrying Scenarios in Genetic Discrimination—Based on Questionnaires from China

**DOI:** 10.3390/healthcare11020188

**Published:** 2023-01-08

**Authors:** Zhong Wang, Yujun Guo, Rui Xu

**Affiliations:** 1 School of Economics, Guangdong University of Technology, Guangzhou 510520, China; 2 Key Laboratory of Digital Economy and Data Governance, Guangdong University of Technology, Guangzhou 510520, China; 3 LIESMARS, Wuhan University, Wuhan 430079, China

**Keywords:** genetic discrimination, attitude disparity, genetic testing, worrying scenarios, genetic data application

## Abstract

**Objectives:** As genetic testing is increasingly used in non-medical fields, the judgment of people’s potential conditions based on predictive genetic information inevitably causes genetic discrimination (henceforth GD). This article aimed to systematically investigate the disparity in attitudes and worrying scenarios concerning GD in China. **Methods:** A questionnaire survey of 555 respondents was conducted. Statistical tests were used to examine disparity in attitudes between gender, age, and education. A descriptive analysis was also conducted to explore other worrying scenarios. **Results:** It shows that (1) men are more tolerant of GD compared to women, and (2) participants aged between 18 and 30 years old possess the highest objection to GD. However, (3) no indication can attest to the relationship between educational level and perspective on GD. In addition, (4) the acceptance of gene testing in the three most common scenarios is ranked in descending order as follows: partner choice, insurance services, and recruitment. Moreover, (5) worrying scenarios relating to GD include: education, social occasions, medical services, fertility, shopping, and so on. **Conclusions:** Based on the results, suggestions proposed include developing a blacklist mechanism in the field of genetic data application and strengthening the security regulations for the commercial use of genetic data.

## 1. Introduction

In line with the development of gene technology since the 1980s [1,2], the usage of genetic data has become more extensive, which has gradually intensified concerns about genetic discrimination (henceforth GD). Genetic technology can be applied to many domains, such as criminal investigations [3], personalized medical services and clinical treatments [4], and genetic risk prediction [5]. Some individuals have declined genetic testing due to fears about insurance discrimination based on gene defects [6]. GD has become a more common issue relating to genetic research and brings both social and psychological problems [7]. GD refers to the differential treatment [8] given to those people who have apparent or perceived ‘abnormal’ variations in their genetic information compared with the ‘normal’ human genotype [9]. It is an adverse selection based on genetic information [10].

Recruitment [11], insurance services [12], and partner choice [13] are the three circumstances widely studied for exploring discrimination associated with genetic information. Firstly, employers might ask for genetic information to evaluate and predict the potential abilities and health conditions of prospective employees. Sometimes, employers might regard genetic information as a precondition for initial employment, the continuance of employment, or promotion [10], and there are indeed cases where individuals were disqualified or dismissed due to their abnormal genotypes [9]. Based on private genetic information, differential treatment in the workplace, including promotions, would be adopted to maximize profits and minimize the cost of compensation associated with healthcare [10]. Some countries have taken measures (e.g., the U.S. [14,15], Canada [16]), while others still have gaps (e.g., China). In China, there are currently some research foci on gene equality as well as legislative protection for the genetic information of employees [17]. Understanding public acceptance of levels of discrimination related to genetic information is extremely important for improving the rational usage of genetic information in the hiring process. Secondly, genomic stratification and risk classification exist, which cause debate about GD in the context of life insurance [18]. The insurer might inappropriately use private healthcare information, limiting the coverage of insurance, rearranging the coverage by increasing insurance rates [18], or adjusting the contents of insurance if the genotype of consumers indicates a risk of severe diseases. In some cases, vital services might be denied [19] by unfair considerations of genetic information. Most anti-GD policies usually focus on eliminating GD in the field of public health insurance. In contrast to public health insurance, private insurance is more of a commercial product, which is harder to control but also deserves rigorous supervision. Thirdly, genetic counselling before partner choice or pregnancy is popular, especially in countries possessing a high occurrence of customary consanguineous marriage [20]. Gene testing helps to identify the mutations that underlie recessive disorders or those that might cause psychiatric problems [21,22]. However, people with abnormal genome types are worried about being excluded from consideration as a candidate for marriage. Nowadays, genetic matching for marriage is emerging in many dating applications or websites in China, such as Jiayuan.com [23]. However, these might cause GD. People’s attitudes towards the disclosure of genetic information during partner choice have not yet been well discussed. Additionally, genetic information is also applied in other fields, such as precision medicine [24], criminal investigations, and so on. Disease predisposition [25] and personalized medication [26] achieved by utilizing genetic data have become quite popular in the global medical industry in recent years. Underlying the context of enhanced predictive capacities and healthcare costs, more attention should be paid to the protection of groups that are vulnerable to GD. However, it is critical to first understand the general attitude of the public towards GD and gene testing, which is the main purpose of this article.

Measures and regulations have been used by some countries to address the issues associated with GD. Research shows that North America and Europe are the regions with extensive policy-making activities, while Asia has moderate policy-making activities related to GD [27]. In 2008, the Genetic Information Non-discrimination Act was passed at the federal level in the U.S., while in 2010, a survey called the Behavioral Risk Factor Surveillance System was conducted to better assess people’s interest in attending gene testing, concerns about GD in the decision of life insurance eligibility and cost, as well as the need for non-discrimination laws in the context of life insurance [28]. Strict and broad prohibitions against the inappropriate usage of genetic information are expected to reduce the extent of GD [29]. It is noteworthy that the laws and regulations prohibiting genetic disability discrimination are deficient in China [30]. Therefore, there is an urgent need for relevant research to support the formulation or improvement of regulations on GD in China.

Previous research shows that Chinese people were willing to explore using genetic information for disease prevention, while there was less awareness of the side effects (i.e., possible negative effects) [31]. Nowadays, China has become a crucial player in genetics and genomics [32]. Disparities between cultural traditions and institutional backgrounds lead to differences in attitudes and concepts about genetic information between countries. Although there is some literature about GD, the research on GD in China is insufficient, with only a few studies on forensic genetics in China seeming to indicate GD [33]. Moreover, those studies have several limitations. Firstly, the lack of both sufficient evidence and robust methodology has degraded the results of GD research [34]. Secondly, the diversity of the sample population is deficient since the survey objects are only genetic providers in the research for genetic-associated ethical issues in China [35]. Additionally, genetic testing has become a buyer’s market [36] because of the falling price and the fierce competition among suppliers [37]. Considering the limitations listed above and the important role that China plays in the whole gene industry, a systematic study on GD in China is urged. Some questions await answers. *What are the differences in the attitude towards GD among diverse types of people in China? Which scenarios of GD are of concern to Chinese people?* In an attempt to answer these questions, we conducted an online questionnaire survey to explore the disparities between attitudes and worrying scenarios of GD and to propose some suggestions for balancing concerns about GD with technology development.

The paper is structured as follows: Section 2 presents the methodology and data. Section 3 analyzes the results. Section 4 analyzes the reasons for the disparities in attitudes towards GD and discusses the contributions and limitations of this research. In Section 5 we discuss the findings of this study and future research lines.

## 2. Materials and Methods

### 2.1. Survey Development

To systematically study GD concerns in China, an anonymous questionnaire survey was conducted from 27 May to 29 September in 2019. The questionnaire is hosted online by the “Jinshuju” website (https://jinshuju.net (accessed on 1 May 2022), a professional survey company), and was distributed through social media platforms QQ and WeChat developed by Tencent. Tencent’s 2020 financial results show that QQ has 693.5 million monthly active accounts on smart terminals, and WeChat has 1.225 billion monthly active accounts on smart terminals.

A snowball method was applied, and the questionnaire URL had been sent to several WeChat groups and QQ groups by the first author. Then, the members were invited to fill out the questionnaire or forward it to other QQ groups or WeChat groups voluntarily. Participants were informed that their involvement was voluntary and agreed to participate upon completing the questionnaire. Their anonymous responses were used only for research purposes. Electronic informed consent was obtained from all participants before commencing the survey through QQ and WeChat. According to Article 3 and Chapter 1 of the *Regulations for Ethical Review of Biomedical Research Involving Human Beings*, which were issued and implemented by the National Health and Family Planning Commission of the People’s Republic of China in 2016, the study was not within the scope of ethical review. The Academic Committee of Guangdong University of Technology has confirmed this study does not require ethical approval. The guidelines outlined in the Declaration of Helsinki were followed in our study.

There are 14 questions in the questionnaire. In this paper, part of the questionnaire findings are reported (see Supplementary Materials: *Questionnaire*), consisting of four single-choice questions and one open question: (1) participants’ perspectives on GD; (2) participants’ willingness about participating in gene testing for job application related situations; (3) participants’ attitudes about attending gene testing in insurance services; (4) participants’ willingness about conducting gene testing in partner choice; and (5) participants’ understanding of other circumstances that may occur with regards to GD. In addition, three extra questions were used for the collection of demographic information (age, gender, and educational level) about the respondents. The overall setting of the questionnaire is represented in Table 1.

Questions 2 to 4 were designed to investigate the 3 most worrying scenarios for the participants based on the literature. Considering that participants might be negatively impacted by the word “discrimination”, which is a derogatory word in Chinese, the last three questions utilize the word “gene testing” to probe people’s adoption of gene testing in various real-life scenarios, which laterally reflects the respondents’ concerns about GD.

The options of the questionnaire demonstrate the respondents’ views on gene testing and GD directly and are quantified by sequence, as shown in Table 1. The perspectives of the respondents about GD are classified by different scores, ranging from 1 to 4, where 1 means that people think GD is reasonable, while 4 indicates that people think GD is unreasonable. The willingness of respondents to accept gene testing is also scored, where 4 represents their full disapproval of gene testing and 1 means their full approval of gene testing. There is no relationship of inference or explanation between question 1 and questions 2–4 in the *Questionnaire*.

### 2.2. Demographic Information

Up to September 2019, 565 completed questionnaires were received. Ten questionnaires which contained partial or invalid answers were excluded. These 555 replies were regarded as the study sample, which included people with diverse demographic characteristics. Although the sample size is small compared to a population of 1.4 billion, it is a valuable pilot.

A basic demographic analysis of the participants was undertaken. As shown in Table 2, the participation rates are not uniform among the respondents of different ages and educational backgrounds. People aged between 18–45 years old accounted for 91.4% out of the total 555 respondents, while people aged older than 61 represented the remaining 0.7%. As for educational backgrounds, respondents with a bachelor’s degree had higher participation rates (66.5%) than people with a high school diploma or lower (11.4%). People of particular ages and educational groups make more frequent use of social media, so they are weighted heavily in this survey. However, the gender weighting is more equal in contrast.

A higher response rate was obtained from the participants aged 18–45 years old or with higher educational levels. However, the senior (>60) and the groups with a lower educational background did not fully complete or return the questionnaire, perhaps because they are not familiar with gene testing and GD and lack the knowledge to finish the survey. China’s illiterate population accounted for 4.59% of the population aged 15 and above in 2019. Gene testing and GD are unfamiliar to these sections of the population in China. Genetic technologies have increasingly developed only in the last 30 years [6], which goes beyond some people’s knowledge boundaries. The sample of the questionnaire survey covers people of all ages over eighteen, of all genders, and of all education levels in China, and can thus be used for further statistical analysis.

### 2.3. Statistical Method

To statistically investigate respondents’ views on gene testing and GD, a two-step difference analysis was conducted. The specific analyses involved the following three aspects:

#### 2.3.1. Overview of Respondents’ Attitudes on GD

The disparities in attitude towards GD among different demographic groups, which contain factors of gender, age, and educational level, were investigated. Since the information collected was discrete random values and was not normally distributed, the chi-square test was selected, which is widely applied in clinical statistics [38] and suits the analysis of this type of data. The chi-square test with 95% confidence intervals is utilized to identify the difference between the two datasets or among diverse datasets.

#### 2.3.2. Respondents’ Preferences concerning the Application of Gene Testing in Different Scenarios

A significant difference in willingness among participants to conduct gene testing in three real-life scenarios was identified: recruitment, partner choice, and life insurance. When there are two datasets being contrasted, the marginal homogeneity test, which is an extension of the McNemar test for two dependent sample studies, will be used to identify the differences. The Friedman test is used to detect the differences among the above three scenarios.

#### 2.3.3. Respondents’ Cognition of Other Circumstances Where GD Might Occur

A descriptive analysis contrasts the replies to the open-ended question, which was conducted to further explore the respondents’ concerns about other scenarios where GD might occur, the results of which are shown in Section 3.4.

## 3. Results

### 3.1. Overview of the Respondents’ Attitudes on GD

There are 48.6% of the respondents who disapprove of GD and think it is unreasonable. Only 9.4% agree with GD and think it is reasonable. The rest of the respondents (42.0%) hold the opinion that the existence of GD depends on the situation, and their attitude varies depending on the application. To further compare the disparities in people’s views on GD, the specific investigation was undertaken in different demographical groups.

### 3.2. Attitude Disparity on GD in Different Demographical Groups

#### 3.2.1. Attitude Disparity on GD between Genders

The attitude difference on GD between genders is statistically significant with a *p*-value of 0.000 (<0.05), where men are more tolerant of GD than women. As shown in Table 3, the approval rate of GD among the male respondents is 15.2%, while that of the females is 3.3%. However, the disapproval rates of GD in the two are similar.

#### 3.2.2. Attitude Disparity on GD among Different Age Groups

The results show that there is a significant difference among different age groups (*p* = 0.000). Compared to the respondents aged between 18 and 30 years old, the ones aged 31–45 tend to accept GD in real life. The disparity between the two groups is most conspicuous with a *p*-value of 0.000. The approval rate of GD in the 18–30 year old group is 2.2%, while the one in the 31–45 year old group is 13.9% (Table 4). Moreover, 57.7% of the respondents in the former group believe GD is unreasonable, which is the highest disapproval rate of GD among the five age groups.

#### 3.2.3. Attitude Disparity on GD among Different Educational Groups

The differences between the five educational groups were not distinct. People with no formal high school qualifications have a slightly higher rate; with 16.7% thinking that GD is reasonable (see Table 5). Most of the respondents hold the position that GD is unreasonable. It is noticeable that 58.8% of the respondents with a doctorate consider GD an unreasonable thing in real life, which is the highest disapproval rate of GD among the five groups. However, there is no statistical difference among people with different education levels (*p* = 0.802 > 0.05), and there is little evidence to prove the correlation between an individual’s educational level and their perspective on GD according to the test.

### 3.3. Willingness Disparity in Gene Testing in Real-Life Scenarios

Respondents’ concerns about GD are not stereotypical in different contexts. Analysis shows that the acceptance of gene testing in partner choice is highest in the areas of recruitment and insurance services. As shown in Table 6, the approval rate of gene testing reaches 43.6% in partner choice, where only 6.3% and 7.9% of the respondents support gene testing in job- and insurance-related processes, respectively. In addition, the significant disparities between their willingness to attend gene testing in partner choice and in the other two scenarios are proved with the *p*-values of 0.000 and 0.000, respectively, which indicate that the respondents show significantly higher acceptance to attend gene testing in partner choice when compared with the contexts of recruitment and life insurance. Therefore, it can be concluded that partner choice is the area where the respondents are most inclined to adopt gene testing out of these three key scenarios.

### 3.4. Investigation of the Respondents’ Concerns about Other At-Risk Circumstances

**Figure 1:**
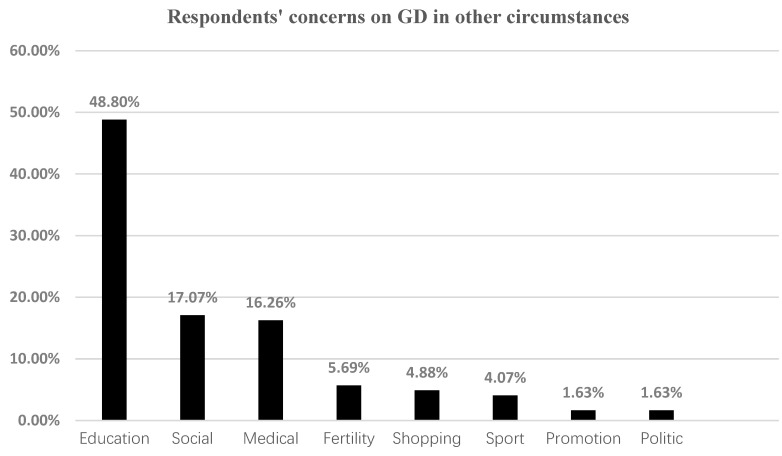
The respondents’ concerns about GD in other circumstances.

Respondents’ understanding of other circumstances where GD might occur includes education, social, medical, and so on. Respondents were required to write down other scenarios in which they thought GD might occur. Some of the respondents left the answer blank or repeated one of the three scenarios studied above. Only 121 replies were valid in answering the question, among which two respondents gave two answers; thus, there are 123 valid answers in total for this question. The composition of the answers collected is shown in Figure 1. The percentage of respondents who were concerned about genetic discrimination in education was as high as 48.8%.

## 4. Discussion

Based on the above statistics on the disparity between respondents’ views on GD and gene testing, several analyses of the reasons for these disparities were conducted. Then, we discuss the contributions and limitations of this paper.

### 4.1. Possible Reasons for Attitude Disparity on GD

#### 4.1.1. Women Maybe More Concerned about the Side Effects of GD

Women have more reservations about discrimination based on genes. On the one hand, it is probably because women are the ones who get pregnant and give birth, which to some extent influences their willingness to undergo gene testing. On the other hand, men, who tend to focus more on promotion and earning money for the family, might be more concerned about the health indications derived from gene testing to help them better prevent potential diseases. It might also be attributed to the willingness to accept innovation, where men show more interest in new technologies [39], while women are probably more anxious about the side effects brought by modern techniques.

#### 4.1.2. The Younger Group Is Inclined to Show More Sensitivity and Opposition about GD

The knowledge of genes is updated very quickly, and many of its contents have not been studied systematically by previous students. Thus, people in the older group might have been less well educated about the knowledge of genes and GD when they were students. Moreover, people aged 31–45 years are more mature and likely more sophisticated on such discriminations. As a result, the younger age group is inclined to show more sensitivity and opposition toward GD.

#### 4.1.3. More Educated People Pay More Attention to the Negative Effects of GD

Among respondents with different educational backgrounds, those with a doctoral degree have the highest disapproval rate for GD. This might be attributed to the abundant educational experiences providing them with more opportunities to access relevant research and experiments, which may emphasize the negative effects of GD.

#### 4.1.4. Most of the Respondents Worry about Job- or Insurance-Related Discrimination

Through the respondents’ acceptance of genetic testing in different scenarios, concerns about GD are reflected. In China, prenatal genetic diagnosis by gene sequencing [40] and gene counselling [41] is common. Genome sequencing is used to make genetic diagnoses in critically ill infants with a rapid turnaround time [42]. People who recognize the benefits of genetic tests in promoting family happiness and protecting offspring health, might be more interested in genetic testing and counselling before marriage or pregnancy. However, concerns over GD in recruitment and insurance services are more severe due to the potential risk of it being harmful to their future careers and job applications. Another consideration is the disclosure of genetic information to third parties, which might lead to malignant privacy infringements. Therefore, due to worries about job- or insurance-related discrimination [20], most of the respondents are opposed to using genetic testing in these two scenarios.

### 4.2. Concerns about GD in Educational, Social, Medical, and Other Scenarios

According to the statistical results, discrimination in education is of the greatest concern to the respondents. The concerns in this area mainly focus on the school admissions-related process. Competition for admission to basic education and higher education in China is fierce. The prevalence of genetic testing poses a high risk of unfairly depriving predisposed students of their rights and opportunities to receive basic or higher education. Such discrimination based on gene testing information may result in obvious inequities in education [43], which has sparked considerable controversy among the public.

GD on social occasions is of great concern to the respondents. The discrimination based on genetic information may lead to vicious isolation in daily meetings. Persons with abnormal or criminal genetic composition are at risk of being ostracized by society or being exposed to violent treatments [44]. The stress and negative effects brought about by discrimination are harmful to individuals’ physical and psychological health.

Medical treatment is the third controversial scenario where GD occurs frequently. Since the 1990s, insurance has been the primary concern for the general public due to adverse selection based on genetic test results [45]. Some medical institutions would categorize and prioritize patients based on their genetic information rather than the sequence of their arrival, and some of them would even reject treating such patients to avoid a negative effect on their reputation. Indeed, the usage and dissemination of genetic data is of great concern to the patients. Customized, efficient medicines and therapies based on personal genetic information have become popular in recent years and are the main direction of medical developments in the future. With the development of technology, the disclosure of private genetic information is hard to avoid. However, disclosure might result in the abuse of such information and GD afterwards [46], which might be the main reason for the respondents’ concerns about the wrong usage and improper dissemination of their private genetic information. All of these concerns deserve urgent attention by the public when conducting rigorous management of the development of genetic technology and the use of genetic data.

Worries about GD in other real-life scenarios such as fertility, leisure activities, and the sports industry have also been put forward by the respondents. The conduction of genetic testing gives rise to inequality by depriving certain groups of people of their pregnancy and fertility rights. In addition, artificial interference in genetic information such as genetic editing in embryos is of great concern to the public [47]. Some respondents probably think that this issue is at risk of intensifying social polarization in China.

Shopping is another field where genetic data is widely used. Businesses analyze consumers’ genetic data to price differently and customize discriminatory services based on their preferences. In the sports industry, the prevalence of gene testing poses a negative impact on justice [48]. The selection might be highly influenced by the genetic information of the athletes rather than their daily performance evaluation, which is arbitrary and might impede the development of people with genetic defects but who are experienced and perform excellently.

Some respondents also state the influence of GD in personal career promotions. The advancement of their careers might be negatively influenced by the consequences of gene testing. Since employers might seriously consider the talent development cost as well as the potential risk based on the predictive health condition of their staff, they are more inclined to select the individual who has proven higher talent and “safety”. In addition, genetic testing might be used by employers to select employees who will always be at work every day and potentially always be available to work. Many people may lose job opportunities [49].

As for their concerns about the effect of GD on politics, people worry that the division of parties would be determined by personal genetics. People with similar genetic compositions would aggregate together, which is bad for national and ethnic solidarity.

According to all the concerns stated above, the wider application of gene testing deserves wider attention and careful consideration. Genetic technology can benefit our lives only if the data has been applied appropriately and scientifically. Furthermore, consistent control of the occurrence of GD, as well as the prevention of privacy infringement, is of great necessity to establish a better environment for the developments of genetic technology in society.

### 4.3. Theoretical Contributions

By answering the research question, “What are the differences in the attitudes of GD between different crowds of people in China? What are the scenarios of GD concern by the Chinese?” we claim as theoretical contributions to the wider discussion of GD literature. Our major contribution to GD literature includes: (1) having analyzed the attitudes of different groups in China towards GD; (2) having analyzed attitude disparities between three key scenarios, namely, insurance, partner choice, and recruitment; and (3) having explored other scenarios where Chinese people worry that GD might exist and impact their lives. This is a significant basis for follow-up related research, such as genetic technology acceptance, genetic technology diffusion, and gene industry research, especially as China is a major player and has the largest market in this field. Existing studies have put forward the idea that different groups have different concepts of GD, but few studies have been verified from an empirical perspective. This paper investigated it in the form of a questionnaire survey.

### 4.4. Practical Implications

The observations generated in our analysis also have practical implications for genetic technology and industry development. This study provides policy enlightenment not only for China but also for other countries. China has always upheld a relatively open attitude towards genetic technology. To some extent, the case of He Jiankui, a Chinese biophysics researcher who edited the genes of twin babies and was jailed for 3 years [47], caused the regulatory authorities to tighten supervision over genetic technologies. Chinese people’s concerns about scenarios where GD might exist negatively are of referential value for technology developers, industrial circles, and other countries or regions. Specifically, the policy implications of this paper are summarized below.

#### 4.4.1. Develop a Blacklist Mechanism in the Field of Genetic Data Application

Excessive faith in the predictions of genetic information leads to discrimination, bringing social and psychological risks [50] to individuals who are asymptomatic but pre-diagnosed by gene testing. To better regulate the usage of gene data and avoid the occurrence of discrimination, a blacklist mechanism (i.e., a list of scenarios that should prohibit the use of genetic testing) should be implemented for industries with high risks of genetic data abuse and GD. Regulations are needed to restrict the usage of genetic data in these listed areas, and discriminatory treatments should be prohibited to avoid the occurrence of infringement and inequality.

Nowadays, using genetic data for differential pricing in the insurance field in China has been banned. Similar systems should be set up in other fields as well. For instance, in order to protect labor rights, the application of genetic data should be prohibited in the hiring process; in order to maintain education fairness, the application of genetic data should be prohibited in the educational field; in order to avoid social isolation, private genetic data should not be open to the public; in order to protect personal medical data from being accessed and to avoid unfair treatment of patients, the management of medical gene data should be stricter; and in order to guarantee fair competition, sports competitions should not use genetic data to select players. To safeguard the diversity of the human gene bank, gene screening at the reproductive stage should be regulated, lest the excessive use of genetic data bring devastating disaster to human beings.

#### 4.4.2. Strengthen the Security Regulation for the Commercial Use of Genetic Data

Enacting regulations and laws to restrict the inappropriate use of genetic information and safeguard personal privacy is imperative. In order to promote industrial self-discipline and integrity, first, ethical guidelines on the application of gene testing are urged by Chinese geneticists for the improvement of genetics services in China, as well as filling the cultural gap between China and Western countries to reach an agreement on the ethical, legal, and social issues of genetics in the future [51,52].

Initiating genetic non-discrimination laws would protect the public from social discrimination and ease their concerns about GD, thereby enabling them to benefit from genetic technologies [53]. In the past, only the laboratories of large scientific research institutions could carry out genetic data analysis and processing, and related policies and regulations were mainly formulated for scientific research. Nowadays, many enterprises are also able to carry out this work, so the level of information security level should be a barrier to entry. Regulations should clarify the requirements of commercial organizations’ information security levels concerning genetic data protection in order to prevent information safety accidents.

### 4.5. Limitations

Inevitably, there are some deficiencies in this research. Firstly, the representativeness of the sample is not guaranteed. The format of an online questionnaire survey excludes some groups of peoples, such as the elderly and those who are not able to conveniently access the Internet. This affects the representativeness of sampling. China has a large population that does not use social media. Secondly, the questionnaire design was not sufficiently refined to allow for explanatory research. This study mainly describes and analyzes the differences in attitudes towards GD among people of different genders, ages, and educational levels. It has not yet analyzed in depth the reasons for the differences. Additionally, the income data was not included in the design of the questionnaire since it is so sensitive that it may cause some respondents to be unwilling or unable to complete the questionnaire. What’s more, open-ended questions were posed for the study of other scenarios. This makes variance analysis impossible, which is significant for determining the different views of different groups.

## 5. Conclusions

Through the form of a questionnaire survey, this study has analyzed different attitudes of various groups towards GD in the scenarios of partner choice, recruitment, and insurance purchase, as well as other real-life scenarios where Chinese people worry that GD might exist. To facilitate the benign development of industrial ecology, some pieces of policy advice are also suggested. 

With regard to future research, some in-depth research can be conducted. For example, insurance can be divided into health and life insurance, and thus a differentiation study can be conducted. Regarding partner choice, an in-depth interview can be carried out to study the causes of differences in views among different groups. What’s more, when it comes to specific scenarios, fairness in education, which is of great concern to the Chinese people, needs a special study in the future.

## Figures and Tables

**Table 1:** The overall setting of the survey.

	Questions	Options	Score
**Normal Questions**	1. What do you think of GD?	Unreasonable	4
		Reasonable in a few cases	3
		Reasonable in most cases	2
		Reasonable	1
	2. What do you think of the application of gene testing during recruitment?	Disapproval	4
		Approval in a few cases	3
		Approval in most cases	2
		Approval	1
	3. What do you think of the application of gene testing during insurance services?	Disapproval	4
		Approval in a few cases	3
		Approval in most cases	2
		Approval	1
	4. What do you think of the application of gene testing in partner choice?	Disapproval	4
		Approval in a few cases	3
		Approval in most cases	2
		Approval	1
	5. Under what other circumstances do you think GD might occur?	Open-ended	Null

**Table 2:** Basic demographic analysis of the participants.

Demographic Information	Classification	Quantity	Rate
Gender	Male	282	50.8%
	Female	273	49.2%
	18–30	227	40.9%
Age	31–45	280	50.5%
	46–60	44	7.9%
	>60	4	0.7%
Educational Level	No formal high school qualifications	12	2.2%
	High school/vocational education level	51	9.2%
	Bachelor’s degree	369	66.5%
	Master’s degree	89	16.0%
	Doctor’s degree	34	6.1%

**Table 3:** Statistical analysis of gender differences in GD attitudes.

Attitude	Male		Female	
	Quantity	Rate	Quantity	Rate
Unreasonable	135	47.9%	135	49.5%
Reasonable in a few cases	86	30.5%	126	46.2%
Reasonable in most cases	18	6.4%	3	1.1%
Reasonable	43	15.2%	9	3.3%
Total	282	100%	273	100%
Confidence Interval	95%			
Significance (*χ*^2^) ^a^	0.000			
H_0_	There is no significant attitude difference towards GD between genders.			
Conclusion	Reject H_0_: There is a significant attitude disparity towards GD between genders, where the males have a higher tendency to accept GD.			

^a^ The chi-square test was conducted for calculating the significance level which was used to identify the difference between two groups. If significance level < 0.05, there is significant disparity between the two.

**Table 4:** Statistical analysis of attitude disparity on GD among different age groups.

Attitude	18–30 (2)			31–45 (3)			46–60 (4)			>60 (5)		
	Quantity	Rate		Quantity		Rate	Quantity	Rate		Quantity		Rate
Unreasonable	131	57.7%		116		41.4%	21	47.7%		2		50%
Reasonable in a few cases	87	38.3%		111		39.6%	12	27.3%		2		50%
Reasonable in most cases	4	1.8%		14		5.0%	3	6.8%		0		0
Reasonable	5	2.2%		39		13.9%	8	18.2%		0		0
Total	227	100%		280		100%	44	100%		4		100%
Confidence Interval	95%											
Significance(*χ*^2^) ^a^	0.000											
H_0_	People with different ages hold similar attitude towards GD.											
Conclusion	Reject H_0_: There is significant attitude disparity among different age groups.											
	2 vs. 3		2 vs. 4		2 vs. 5		3 vs. 4		3 vs. 5		4 vs. 5	
Significance(*χ*^2^) ^a^	0.000		0.000		0.799		0.278		0.275		0.204	
Conclusion	Different		Different		Similar		Similar		Similar		Similar	

^a^ The chi-square test was conducted for calculating the significance level which is used to identify the difference between two groups or among diverse groups. If significance level <0.05, there is significant disparity among the groups.

**Table 5:** Statistical analysis of attitude disparities on GD among different educational levels.

Attitude	No Formal High School Qualifications (1)		High School/Vocational Education Level (2)		Bachelor’s Degree (3)		Master’s Degree (4)		Doctor’s Degree (5)	
	Quantity	Rate	Quantity	Rate	Quantity	Rate	Quantity	Rate	Quantity	Rate
Unreasonable	3	25.0%	25	49.0%	186	50.4%	36	40.4%	20	58.8%
Reasonable in a few cases	7	58.3%	19	37.3%	134	36.3%	40	44.9%	12	35.3%
Reasonable in most cases	0	0	2	3.9%	16	4.3%	3	3.4%	0	0
Reasonable	2	16.7%	5	9.8%	33	8.9%	10	11.2%	2	5.9%
Total	12	100%	51	100%	369	100%	89	100%	34	100%
Confidence Interval	95%									
Significance (*χ*^2^)^a^	0.802									
H_0_	People with different educational levels hold similar attitudes towards GD.									
Conclusion	Accept H_0_: There is no significant disparity in attitudes among different educational groups.									
	1 vs. 2		1 vs. 3		1 vs. 4		1 vs. 5		2 vs. 3	
Significance (*χ*^2^) ^a^	0.787		0.671		0.783		0.330		0.800	
Conclusion	Similar		Similar		Similar		Similar		Similar	
	2 vs. 4		2 vs. 5		3 vs. 4		3 vs. 5		4 vs. 5	
Significance (*χ*^2^)	0.991		0.290		0.735		0.283		0.247	
Conclusion	Similar		Similar		Similar		Similar		Similar	

^a^ The chi-square test was conducted for calculating the significance level which is used to identify the difference between two groups or among diverse groups. If significance level <0.05, there is significant disparity among the groups.

**Table 6:** Statistical analysis of attitude disparities on gene testing among different scenarios.

Attitude	Recruitment (1)		Life Insurance (2)		Partner Choice (3)	
	Quantity	Rate	Quantity	Rate	Quantity	Rate
Disapproval	149	26.8%	148	26.7%	138	24.9%
Approval in a few cases	351	63.2%	344	62.0%	0	0
Approval in most cases	20	3.6%	19	3.4%	175	31.5%
Approval	35	6.3%	44	7.9%	242	43.6%
Total	555	100%	555	100%	555	100%
H_0_	People hold similar attitudes towards gene testing in different scenarios.					
Confidence Interval	95%					
Significance (Friedman test) ^a^	<0.001					
Conclusion	Reject H_0_: There is significant attitude disparity among different scenarios.					
	1 vs. 2		1 vs. 3		2 vs. 3	
Significance (marginal homogeneity test) ^b^	0.345		0.000		0.000	
Conclusion	Similar		Different		Different	

^a^ Friedman test was conducted for calculating the significance level which is used to identify the difference among diverse groups. If the significance level < 0.05, there is significant disparity among the groups. ^b^ Marginal homogeneity test (two-tailed) was conducted for calculating the significance level which is used to identify the difference between two groups. If the significance level <0.05, there is significant disparity between the two.

## Data Availability

The published article includes all datasets generated or analyzed during this study. The data are included as electronic supplemental material in a spreadsheet format (.xlsx).

## Supplementary Materials

The following supporting information can be downloaded at: https://www.mdpi.com/article/10.3390/healthcare11020188/s1, Table S1: Questionnaire; *Categorized data; Statement that no ethical review is required*.
