# Supplementary material for: Attitude Disparity and Worrying Scenarios in Genetic Discrimination—Based on Questionnaires from China
Source: Healthcare (Basel). 2023 Jan 8;11(2):188. doi: 10.3390/healthcare11020188 (PMC9859512; doi:10.3390/healthcare11020188)
Supplement: Supplementary file 1 [file healthcare-11-00188-s001.zip › Questionnaire.pdf]

Questionnaire

Genes support the basic structure and performance of life, and store all kinds of information about life. With the continuous advancement of technology, the cost of obtaining genetic data is decreasing, the scope of use is expanding, and the risk of people's privacy leakage is also increasing. The purpose of this study is to investigate people's knowledge and attitudes towards genetic privacy in order to improve corresponding protection mechanisms. The questionnaire is completed voluntarily by you and the relevant data are for research purposes only. We sincerely thank you for your support and cooperation!

Questions with a star indicate required answers, and those without a star indicate optional answers.

Normal Questions

1. What do you think of genetic discrimination? *

☐ Unreasonable

☐ Reasonable in a few cases

☐ Reasonable in most cases

☐ Reasonable

2. What do you think of application of gene testing during recruitment? *

☐ Disapproval

☐ Approval in a few cases

☐ Approval in most cases

☐ Approval

3. What do you think of application of gene testing during insurance services? *

☐ Disapproval

☐ Approval in a few cases

☐ Approval in most cases

☐ Approval

4. What do you think of application of gene testing in partner choice? *

☐ Disapproval

☐ Approval in a few cases

☐ Approval in most cases

☐ Approval

5. What other circumstances do you think GD might occur?

Open-ended.

Basic demographic questions

1. Gender? *

☐ Male

☐ Female

2. Age? *

☐ 18-30

☐ 31-45

☐ 46-60

☐ >60

3. Educational Level? *

☐ No formal High School qualifications

☐ High school/vocational education level

☐ Bachelor's degree

☐ Master's degree

☐ Doctor's degree

Note

Finally, a dialog box pops up when participants submit the questionnaire: The data you submitted is only for scientific research, do you confirm the submission?
