# Supplementary material for: Attitude Disparity and Worrying Scenarios in Genetic Discrimination—Based on Questionnaires from China
Source: Healthcare (Basel). 2023 Jan 8;11(2):188. doi: 10.3390/healthcare11020188 (PMC9859512; doi:10.3390/healthcare11020188)
Supplement: Supplementary file 1 [file healthcare-11-00188-s001.zip › Statement that no ethical review is required.pdf]

Statement that no ethical review is required

We have reviewed Dr. Zhong Wang's paper and concluded that it does not require ethical review. The paper titled "Attitude Disparity and Worrying Scenarios in Genetic Discrimination--Based on Questionnaires from China" used a questionnaire research method, which was distributed online and completed voluntarily and anonymously by respondents. The survey questions included: people's views on genetic discrimination, worrying scenarios on genetic discrimination, and their views on genetic testing in insurance, employment, and marriage scenarios. According to Article 3, Chapter 1 of the **Regulations for Ethical Review of Biomedical Research Involving Human Beings,** which was issued and implemented by the National Health and Family Planning Commission of the People's Republic of China in 2016, the study was not within the scope of ethical review.

The Article 3, Chapter 1 of the regulations mentioned above states that *ethical review of biomedical research involving humans includes the following activities: (1) the use of modern physics, chemistry, biology, Chinese medicine and psychology and other methods of human physiology, psychological behavior, pathological phenomena, disease etiology and pathogenesis, as well as the prevention, diagnosis, treatment and rehabilitation of disease research activities; (2) new medical technology or new medical products in the human body for experimental research activities; (3) the use of epidemiological, sociological, psychological and other methods of collection, recording, use, reporting or storage of scientific research information about human samples, medical records, behavior, etc.*

The original Chinese text of the law is as follows. 2016 年国家卫生和计划生育委员会颁布实施的《涉及人的生物医学研究伦理审查办法》第一章第三条：*涉及人的生物医学研究伦理审查包括以下活动：(1) 采用现代物理学、化学、生物学、中医药学和心理学等方法对人的生理、心理行为、病理现象、疾病病因和发病机制，以及疾病的预防、诊断、治疗和康复进行研究的活动；(2) 医学新技术或者医疗新产品在人体上进行试验研究的活动；(3) 采用流行病学、社会学、心理学等方法收集、记录、使用、报告或者储存有关人的样本、医疗记录、行为等科学研究资料的活动。*

XIE Weihong

Deputy Director of Academic Committee, Guangdong University of Technology

TEL: +86-020-87082461 EMAIL：xwh513@gdut.edu.cn
